# Frailty is Associated With Poor Outcomes Following Emergency Laparotomy: What’s Next?

**DOI:** 10.7759/cureus.27071

**Published:** 2022-07-20

**Authors:** Sofian Youssef, Ameen Chekroud, Amit Shukla, Milind Rao

**Affiliations:** 1 Academic Research, University of Nottingham, Nottingham, GBR; 2 General Surgery, Nottingham University Hospitals NHS Trust, Nottingham, GBR; 3 General Surgery, Lincoln County Hospital, Lincoln, GBR; 4 General Surgery, Pilgrim Hospital Boston, Boston, GBR

**Keywords:** cfs, emergency, laparotomy, clinical frailty scale, frailty

## Abstract

Background and objective

The Clinical Frailty Scale (CFS) is a rapid assessment tool to identify vulnerable and frail patients. We sought to evaluate the association between preoperative CFS scores and outcomes following emergency laparotomy in a dense, rural, and healthcare-deprived region of the UK inhabited by a multi-comorbid population.

Methods

We retrospectively reviewed regional National Emergency Laparotomy Audit (NELA) data across United Lincolnshire Hospitals NHS trust to identify all patients aged 65 years and above who underwent emergency laparotomy between December 2018 and March 2021. We also conducted a comprehensive multi-database literature search of Medline, Embase, and Cochrane to synthesise contemporaneous topical evidence.

Results

A total of 191 patients were assessed using the CFS before they underwent emergency laparotomy. Among 90 (47.1%) individuals categorised as vulnerable or frail (CFS score ≥4), there was no significant difference in age, gender, or length of stay related to the procedure compared with fit patients. However, vulnerable and frail patients were significantly more likely to die (84.8% vs. 39.2%, p<0.0001). Regression analysis identified a vulnerable or frail score to be a significant predictor of 30-day all-cause mortality (OR: 9.327; 95% CI: 3.101-28.054; p<0.0001). A total of six relevant papers were identified in the literature, all indicating a significant association between mortality as well as prolonged length and stay with clinical vulnerability and frailty.

Conclusions

The CFS is a practical and effective tool for assessing preoperative vulnerability and frailty among patients undergoing emergency laparotomy and can be used to predict mortality and morbidity after surgery.

## Introduction

Risk-benefit evaluation is a fundamental process used by surgeons in deciding when to operate and when not to operate. The decision to perform a surgery still varies considerably due to different factors [1-3]. Objective assessment and risk stratification tools such as the National Emergency Laparotomy Audit (NELA) and Portsmouth Physiological and Operative Severity Score for the enumeration of Mortality (P-POSSUM) have improved the process [4]. However, the lack of universal, comprehensive guidelines regarding their practical use has meant that decisions to operate in complex cases are still largely subjective. An important consideration in such situations is the long-term quality of life for patients. This has recently been addressed with the introduction of the Clinical Frailty Scale (CFS) [5].

Frailty is defined by the World Health Organisation (WHO) as a clinically recognisable state, in which the ability of older people to cope with everyday or acute stressors is compromised by age-related decline in physiological reserve and function across multiple organ systems [6]. Frailty is estimated to affect 6.5% of those aged 60-69 years, and 65% of individuals over the age of 90 [7]. While the gradual decline in functional ability seen in frailty is not linear or constant, a frail individual is more likely to deteriorate over time [8]. The frailty phenotype arose as a measure of frailty in a clinical context based upon grip strength, walking speed, activity levels, self-reported energy or exhaustion, and unintentional weight loss [9]. Numerous methods of characterising frailty have since been developed; an emerging, increasingly popular, rapid, and easy-to-use method of indicating frailty is the CFS. In recent years, the 9-point CFS has proven effective in surgical settings to predict outcomes, such as mortality and long-term functionality [5,10] (Appendix 1).

The impact of frailty on outcomes in healthcare has been an ongoing area of cross-specialty interest in order to utilise resources effectively. This also prevents unnecessary harm by implementing the appropriate course of treatment for the patient. Meta-analyses have shown that increasing frailty based on the CFS is associated with higher hospital mortality and poorer quality of life in intensive care settings [11], with some studies showing an incremental increase in risk with increasing CFS [12]. There is increasing evidence that frailty is associated, in a predictable fashion, with postoperative complications irrespective of the procedure [13,14], and within emergency general surgery [15]. The Royal College of Anaesthetists recommends the preoperative assessment of frailty as a good practice in both elective as well as emergency procedures [16]. This can help predict adverse outcomes, ensure optimal perioperative management, and allow for any required preoperative optimisation.

In this study, we aimed to evaluate the use of CFS (clinical vulnerability and frailty) as a predictor of mortality in patients undergoing emergency laparotomy in a rural, ageing, and multi-comorbid population.

## Materials and methods

Study design

We conducted a retrospective observational study by reviewing NELA data to identify patients who underwent emergency laparotomy between December 2018 and May 2021 at the United Lincolnshire Hospitals NHS Trust (ULHT). This trust caters to a population of three-quarters of a million in the East of England.

Setting

We reviewed data from Lincolnshire, a county in the East of England, populated by over 750,000 individuals. Years living with disability (YLD) is a scale used by Public Health England to quantify the burden of morbidity; YLD in Lincolnshire is estimated to be around 15,000 per 100,000 people and is increasing more quickly compared to regional and national rates [17]. The overall burden of disease is also far greater in Lincolnshire as compared to the East Midlands and the rest of the UK [17]. United Lincolnshire Hospitals NHS Trust consists of three separate centres, with Lincoln County Hospital and Pilgrim Hospital performing all emergency surgical procedures.

Data collection

We included all patients aged 65 years and older who underwent emergency laparotomy between December 1, 2018, and May 31, 2021. Patients who had previously undergone surgical procedures during the same admission were excluded. Routinely collected data as part of the NELA data set from December 2018 onwards at our centre included Rockwood’s CFS, P-POSSUM, American Society of Anesthesiologists (ASA) score, NELA score, as well as intraoperative and postoperative outcome variables.

Our primary endpoint was all-cause mortality occurring within 30 days of emergency laparotomy of all kinds. Secondary endpoints included reoperation within 30 days and hospital length of stay from the date of the first procedure. Ethical approval was not required for this study as this was a service evaluation of routinely collected data. Ratification was sought from local auditing departments.

Statistical analysis

Statistical analyses were performed using SPSS Statistics Version 27.0 (IBM Corp., Armonk, NY). Patients deemed as fit (CFS score 1-3) were compared with those considered as vulnerable or frail (CFS score ≥4). Data were analysed for normality using the Shapiro-Wilk test and analysed using descriptive statistics. Continuous variables were compared using an unpaired t-test or Wilcoxon rank sum test depending on variable normality. Categorical variables were compared using a Chi-squared test. A Fisher's exact test was used based on expected cell count if any of the factor-level combinations was less than five. A binary logistic regression model was used to evaluate the significance of the relationship between CFS (fit vs. vulnerable and frail), demographics (age, comorbidities, and BMI), and mortality. Values were presented as OR (95% CI). A p-value <0.05 was considered statistically significant.

## Results

A total of 191 patients were assessed with CFS (Appendix 1), and they underwent emergent laparotomy at United Lincolnshire Hospitals NHS Trust; 101 (52.9%) patients were deemed clinically non-vulnerable (CFS score 1-3) and 90 (47.1%) patients clinically vulnerable or frail (CFS score ≥4). Comparisons between survivors and non-survivors in terms of demographics, validating scoring matrices (CFS, P-POSSUM, NELA, and ASA), and clinical variables are summarised in Table 1.

**Table 1:** A summary of demographic, clinical, and validated scoring matrix variables, comparing survivors with non-survivors within 30 days of emergent laparotomy IQR: interquartile range; CFS: Clinical Frailty Scale; P-POSSUM: Portsmouth Physiological and Operative Severity Score for the enumeration of Mortality; NELA: National Emergency Laparotomy Audit; ASA: American Society of Anesthesiologists

Variables	Total (n=191)	Survivors (n=162)	Non-survivors (n=29)	P-value
Age, years, median (IQR)	75 (72–80.5)	75 (70–80)	75 (72–80.75)	0.463
Male gender, %	56.0%	58.2%	45.5%	0.179
CFS score ≥4, n (%)	90 (47.1%)	65 (40.1%)	25 (86.2%)	<0.0001
P-POSSUM score, median (IQR)	9.6% (4.1%–21.5%)	8.2% (3.9%–16.7%)	22.7% (9.5%–53.9%)	<0.001
NELA score, median (IQR)	9% (4.2%–18.2%)	7.5% (3.5%–13.8%)	23.9% (10.6%–38.4%)	<0.0001
ASA score, median (IQR)	3 (2–3)	3 (2–4)	3 (2–5)	<0.0001
Intensive care unit admission, n (%)	160 (83.8%)	130 (82.3%)	30 (90.9%)	0.221
Systolic blood pressure <90 mmHg, n (%)	12 (6.3%)	9 (5.7%)	3 (9.1%)	0.465

We found clinically vulnerable or frail patients to be at increased risk of 30-day postoperative mortality (41.7% vs. 4%, p<0.0001) and less likely to be returned to theatre for reoperation (3.3% vs. 12.9%, p=0.019). There was no significant difference in terms of the length of hospital stay after the first procedure and ICU admission (Table 2).

**Table 2:** A comparison of all-cause 30-day mortality, return to theatre rates, and length of stay from the date of the first operation between patients deemed fit (CFS score 1-3) and those clinically vulnerable and frail (CFS score ≥4) CFS: Clinical Frailty Scale; IQR: interquartile range

Variables	Fit (CFS score: 1-3)	Vulnerable (CFS score: ≥4)	P-value
All-cause 30-day mortality, n (%)	4 (4.0%)	25 (41.7%)	<0.0001
Reoperation within 30 days, n (%)	13 (12.9%)	3 (3.3%)	0.019
Length of stay, days, median (IQR)	13 (12.6–22.3)	12 (13.4–19.4)	0.713
Intensive care unit admission, n (%)	84 (83.2%)	76 (84.4%)	0.811

Binary logistic regression modeling was used to evaluate the effectiveness of clinical vulnerability or frailty (CFS score ≥4) and validated scoring systems to predict 30-day postoperative all-cause mortality (Table 3). The greatest predictor of mortality was clinical vulnerability and frailty (OR: 9.327; 95% CI: 3.101-28.054; p<0.0001). P-POSSUM (preoperative mortality), NELA, and ASA scores were also identified as significant predictors, although the median ASA score was similar between survivors and non-survivors. The first recorded systolic blood pressure <90 mmHg at presentation to the hospital was not predictive.

**Table 3:** Binary logistic regression for the prediction of mortality in patients undergoing emergency laparotomy CFS: Clinical Frailty Scale; P-POSSUM: Portsmouth Physiological and Operative Severity Score for the enumeration of Mortality; NELA: National Emergency Laparotomy Audit; ASA: American Society of Anesthesiologists

Variables	Odds ratio (95% confidence interval)	P-value
Clinically vulnerable (CFS score ≥4)	9.327 (3.101–28.054)	<0.0001
P-POSSUM score (preoperative mortality)	1.031 (1.014–1.047)	0.0002
NELA score	1.067 (1.034–1.095)	<0.0001
ASA score	3.915 (2.146–7.142)	<0.0001
Male gender	0.625 (0.278–1.407)	0.257
Age	0.985 (0.922–1.052)	0.651
Systolic blood pressure <90 mmHg at admission	0.549 (0.135–2.227)	0.401

## Discussion

Frailty is rapidly emerging as a valuable prognosticator for elderly patients undergoing major surgical interventions. Recent observational data has elucidated CFS as an independent predictor of mortality, length of stay, readmission, and postoperative complications following emergency laparotomy (Table 4) [5,10,18-21]. Research has previously principally investigated frailty (CFS score ≥5), but growing bodies of evidence suggest that clinical vulnerability (CFS score 4) is also a valuable predictor of post-emergency laparotomy outcomes [10,18]. Analysis of our rural trust-wide NELA database showed that a CFS score ≥4 is a significant predictor of postoperative all-cause 30-day mortality (OR: 9.327, 95% CI: 3.101-28.054; p<0.0001). This is consistent with findings from Parmar et al. [10] who performed a national prospective observational study, mainly in the urban centres and obtained the following results: CFS score 4: OR: 7.49 (1.73-32.4), p=0.007; and CFS score 5: OR: 9.79 (2.23-42.91), p=0.002. Our regional data projects Parmar et al.’s findings to a rural, ageing, and multi-comorbid population. To date, all evidence attempting to validate the prognostic utility of CFS has been level 2b or weaker as per the Oxford Centre for Evidence-Based Medicine Levels of Evidence [22]. Ideally, randomised and well-designed research is needed for conclusive validation. Significantly, more research is needed to investigate the steps required to improve the outcomes in these frail and vulnerable patients. McIsaac et al.’s systematic review concluded that few interventions have been tested to improve outcomes and called for higher-quality research in the field [23].

**Table 4:** Summary of evidence evaluating the utility of the CFS in predicting outcomes in patients undergoing emergency laparotomy procedures CFS: Clinical Frailty Scale; HR: hazard ratio; CI: confidence interval; OR: odds ratio

Authors	Title	Definition of frailty (CFS score)	Number of patients/studies	Outcomes					Level of evidence
				1-year mortality	Readmission	Length of stay	30-day mortality	90-day mortality	
Vilches-Moraga et al. [19]	Emergency laparotomy in the older patient: factors predictive of 12-month mortality-Salford-POPS-GS. An observational study	>=5	113	HR: 5.0403 (95% CI: 1.719–16.982), p=0.004	64% (CFS score >=5) vs. 31.7% (CFS score <5), p=0.006	Not studied	Not studied	Not studied	2b
McGuckin et al. [18]	The association of peri-operative scores, including frailty, with outcomes after unscheduled surgery	>=4	164	Not studied	Not studied	Median (IQR): CFS score <4: 9 (6–18) days vs. CFS score >=4: 22 (12–33) days, p<0.001	CFS score <4 [0%] vs. CFS score >=4 [5%], p=0.007	Not studied	2b
Parmar et al. [10]	Frailty in older patients undergoing emergency laparotomy: results from the UK Observational Emergency Laparotomy and Frailty (ELF) study	Investigated at CFS levels 4, 5, and >5	937	Not studied	30-day readmission; OR (95% CI): CFS score 4: 1.93 (0.74–5.04), p=0.18; CFS score 5: OR: 1.16 (0.4–3.37), p=0.78; CFS score >5: OR: 1.22 (0.35–4.19), p=0.75	OR (95% CI): CFS score 4: 1.49 (1.15–1.91), p=0.002; CFS score 5: 1.44 (1.10–1.89), p=0.008; CFS score >5: 1.62 (1.19–2.2), p=0.002	OR (95% CI): CFS score 4: 7.49 (1.73–32.4), p=0.007; CFS score 5: 9.79 (2.23–42.91), p=0.002; CFS score >5: 10.4 (2.24–48.18), p=0.003	OR (95% CI): CFS score 4: 3.15 (1.27–7.84), p=0.014; CFS score 5: 3.18 (1.24–8.14), p=0.016; CFS score >5: 6.1 (2.26–16.45), p<0.001	2b
Carter et al. [20]	Association between preadmission frailty and care level at discharge in older adults undergoing emergency laparotomy	investigated all CFS (on 7-point); frail >=5	956	Not studied	Not studied	CFS score 4 (vulnerable): HR: 0.50 (95 CI: 0.36–0.70), p<0.001; CFS score 5 (mildly frail): HR: 0.52 (95 CI: 0.36–0.77) p=0.001; CFS score 6–7 (moderately or severely frail): HR: 0.55 (95 CI: 0.34–0.88), p=0.013	CFS score >=5: 14.6% (descriptive only, no analysis)	CFS score >=5: 19.5% (descriptive only no analysis)	2b
Alder et al. [5]	Clinical frailty and its effect on the septuagenarian population after emergency laparotomy	>=5	153	Mortality at 19 months: OR: 3.2 (95% CI: 1.09–9.61), p=0.034	Not studied	Not studied	Not studied	Not studied	2b
Arteaga et al. [21]	Impact of frailty in surgical emergencies. A comparison of four frailty scales	>=5, 9-point scale	92	Not studied	Not studied	Not studied	OR: 5.735 (95 CI: 1.453–22.643), p=0.013	Not studied	2b

Almost half (90, 47.1%) of individuals requiring emergency laparotomy regionally in Lincolnshire were characterised as being clinically vulnerable or frail (CFS score ≥4). Despite this, there remain significant discrepancies in the adjusted provision of healthcare facilities across centres in the UK [17]. A collaborative approach among surgeons and frailty teams is needed to assess vulnerable and frail patients. The CFS may be able to provide a quick way of identifying such patients. Despite the available literature, contemporaneous evidence shows that comprehensive frailty assessments by a senior clinician are not routinely offered for patients undergoing major emergency operations [5]. A key barrier to the implementation of joint assessment is the lack and availability of geriatricians and advanced care practitioners during and especially out of working hours. Comprehensive training of surgical decision-makers in conducting frailty assessments is essential to optimise risk-benefit evaluation. Including prognostic data from frailty assessment will also facilitate a more effective discussion regarding informed consent with patients.

Mortality among hypotensive patients undergoing emergency surgery has been extensively researched. Patients with systolic BP <90 mmHg requiring a laparotomy have a reported mortality rate of 46% [24]. Interestingly, we found the first recorded BP at presentation to the hospital to be an insignificant predictor in our regional cohort of patients (Table 3). We also found that the length of hospital stay was insignificantly different between both cohorts of patients (13 vs. 12 days, p=0.713); however, this result is likely skewed by the sizeable difference in 30-day mortality among vulnerable patients. Validated and widely used mortality risk predictors such as P-POSSUM (preoperative mortality risk) and NELA were predictive of mortality (Table 3). Return to theatre was lower among vulnerable and frail patients, which we assume could be due to this cohort of patients having lower chances of survival in reoperation and as such risk-benefit decisions may favour palliative options over the return to theatre.

The proper identification of patients' status can help clinicians appropriately counsel patients and their families and explain the risks. It also helps in weighing up options for surgical vs. non-surgical (palliative) management. The evidence appears to be growing in number and strength, and it has consistently revealed the poor prognosis associated with clinical vulnerability and frailty in patients undergoing emergency laparotomy. However, we are yet to see updated guidelines regarding how we can decrease the complications and improve the outcomes in these patients. This area is in pressing need of further research, which needs to be addressed. Several evidence-based interventions in the pre, peri, and postoperative periods may improve outcomes [25]. Crucially, a constructive, well-organised, and efficient multi-disciplinary approach is required from the moment a patient is assessed as requiring emergency general surgery. This necessitates input not only from surgeons, geriatricians, and physiotherapists but also from specialist nurses in the education of patients, rehabilitation services, as well as dieticians [26].

Limitations

There are some limitations to this study that must be considered. This was a retrospective cohort study with all the known limitations of retrospective data collection. We did not explore all comorbidities, in contrast with what has been done in previous studies, with the intention to focus on variables previously identified as predictors of outcomes and not considered by validated risk predictors such as NELA and P-POSSUM. As mentioned by Rodríguez-Quintero et al. [27], there are an innumerable number of potential confounding variables that affect outcomes following emergency laparotomy, which realistically is impossible to assess in an uncontrolled retrospective study.

## Conclusions

Based on our findings, CFS is effective at predicting emergency laparotomy outcomes in rural, ageing, and multi-comorbid populations. It is a practical and easy-to-use tool that should be formally incorporated into preoperative assessments of those undergoing emergency laparotomy. Identifying patients deemed clinically vulnerable and frail may have implications on managing expectations and obtaining consent prior to emergency surgeries. Further research is required to qualify pre, peri, and postoperative measures to mitigate poor outcomes in vulnerable and frail patients.

## Appendices

**Table 5:** Appendix 1: A summary of key descriptors used in scoring individuals on the Clinical Frailty Scale (CFS)

Score	Frailty classification	Description
1	Very fit	People who are robust, active, energetic, and motivated. These people commonly exercise regularly. They are among the fittest for their age
2	Well	People who have no active disease symptoms but are less fit than individuals who score 1. Often, they exercise or are very active occasionally
3	Managing well	People whose medical problems are well-controlled but are not regularly active beyond routine walking
4	Vulnerable	Although not dependent on others for daily help, symptoms often limit activities. A common complaint is being “slowed up” or being tired during the day
5	Mildly frail	These people often have more evident slowing and need help in high-order instrumental activities of daily living (finances, transportation, heavy housework, medications). Typically, mild frailty progressively impairs shopping and walking outside alone, meal preparation, and housework
6	Moderately frail	People who need help with all outside activities and with keeping house. Inside, they often have problems with stairs and need help with bathing, and might need minimal assistance (cuing, standby) with dressing
7	Severely frail	Completely dependent for personal care, from whatever cause (physical or cognitive). Even so, they seem stable and not at high risk of dying (within ∼6 months)
8	Very severely frail	Completely dependent, approaching the end of life. Typically, they could not recover even from a minor illness
9	Terminally ill	Approaching the end of life. This category applies to people with a life expectancy <6 months who are not otherwise evidently frail
